# BMP2-induced chemotaxis requires PI3K p55γ/p110α-dependent phosphatidylinositol (3,4,5)-triphosphate production and LL5β recruitment at the cytocortex

**DOI:** 10.1186/1741-7007-12-43

**Published:** 2014-05-30

**Authors:** Christian Hiepen, Andreas Benn, Agnieszka Denkis, Ilya Lukonin, Christoph Weise, Jan H Boergermann, Petra Knaus

**Affiliations:** 1 Institute for Chemistry and Biochemistry, Freie Universität Βerlin, 14195 Berlin, Germany

**Keywords:** Actin, BMP, BMP receptor, Chemotaxis, LL5beta, Migration, p110alpha, p55gamma, PHLDB2, PIK3R3

## Abstract

**Background:**

BMP-induced chemotaxis of mesenchymal progenitors is fundamental for vertebrate development, disease and tissue repair. BMP2 induces Smad and non-Smad signalling. Whereas signal transduction via Smads lead to transcriptional responses, non-Smad signalling induces both, transcriptional and immediate/early non-transcriptional responses. However, the molecular mechanisms by which BMP2 facilitates planar cell polarity, cortical actin rearrangements, lamellipodia formation and chemotaxis of mesenchymal progenitors are poorly understood. Our aim was to uncover the molecular mechanism by which BMP2 facilitates chemotaxis via the BMP2-dependent activation of PI3K and spatiotemporal control of PIP3 production important for actin rearrangements at the mesenchymal cell cytocortex.

**Results:**

We unveiled the molecular mechanism by which BMP2 induces non-Smad signalling by PI3K and the role of the second messenger PIP3 in BMP2-induced planar cell polarity, cortical actin reorganisation and lamellipodia formation. By using protein interaction studies, we identified the class Ia PI3K regulatory subunit p55γ to act as a specific and non-redundant binding partner for BMP receptor type II (BMPRII) in concert with the catalytic subunit p110α. We mapped the PI3K interaction to a region within the BMPRII kinase. Either BMP2 stimulation or increasing amounts of BMPRI facilitated p55γ association with BMPRII, but BMPRII kinase activity was not required for the interaction. We visualised BMP2-dependent PIP3 production via PI3K p55γ/p110α and were able to localise PIP3 to the leading edge of intact cells during the process of BMP2-induced planar cell polarity and actin dependent lamellipodia formation. Using mass spectrometry, we found the highly PIP3-sensitive PH-domain protein LL5β to act as a novel BMP2 effector in orchestrating cortical actin rearrangements. By use of live cell imaging we found that knock-down of p55γ or LL5β or pharmacological inhibition of PI3K impaired BMP2-induced migratory responses.

**Conclusions:**

Our results provide evidence for an important contribution of the BMP2-PI3K (p55γ/p110α)- PIP3-LL5β signalling axis in mesenchymal progenitor cell chemotaxis. We demonstrate molecular insights into BMP2-induced PI3K signalling on the level of actin reorganisation at the leading edge cytocortex. These findings are important to better understand BMP2–induced cytoskeletal reorganisation and chemotaxis of mesenchymal progenitors in different physiological or pathophysiological contexts.

## Background

Gradients of bone morphogenetic proteins (BMPs) act as mesenchymal guidance cues during development, disease and tissue repair by molecular mechanisms that remain poorly defined [1]. In particular, the directional migration (chemotaxis) of neural crest cells, bone marrow stromal cells and endothelial cells along gradients of BMP2 has been reported [2-5]. BMPs signal through binding to cell surface hetero-oligomeric receptor complexes comprising type I (BMPRI) and type II (BMPRII) receptors [6]. Activated BMP receptor complexes induce canonical-Smad and non-Smad signalling cascades [7]. Activation of the type I receptor kinase by the type II receptor kinase induces phosphorylation and thus nuclear translocation of Smad1/5/8, leading to transcription of Smad-dependent target genes [8].

Whereas the molecular basis of canonical Smad signalling and its role in gene transcription is well explored, the molecular activation mechanism and the cellular functions of the non-Smad pathways, which rather act directly and independently of gene transcription, are poorly understood. In particular, the molecular mechanism of BMP-induced phosphatidylinositol 3-kinase (PI3K) activation, its signalling route and cellular function are poorly characterised. In recent years, several studies unveiled a requirement of PI3K for BMP2-induced migration of various cell types with mesenchymal origin by yet unknown mechanisms [9-11].

Here, for the first time, we addressed the molecular activation mechanism of BMP2-induced PI3K signalling in undifferentiated mesenchymal progenitor cells and the role of the lipid-product of PI3K, the membrane-bound second messenger PtdIns-3, 4, 5-triphosphate (PI (3, 4, 5) P_3_; hereafter referred to as PIP3) in BMP2-induced actin reorganisation.

Class Ia PI3Ks are dimeric lipid kinases composed of one out of five possible regulatory subunits encoded by *Pik3r1* (encoding splice isoforms p85α, p55α and p50α), *Pik3r2* (p85β) or *Pik3r3* (p55γ) [12,13]. The regulatory subunit is bound by one of three catalytic subunits, termed p110, encoded by *Pik3ca* (p110α), *Pik3cb* (p110β) or *Pik3cd* (p110δ) [14]. Catalytic activity is initiated upon regulatory subunit Src homology 2 (SH2) domain binding to phospho-tyrosine (pTyr) residues within a specific peptide context [15]. Thereafter, activated PI3K phosphorylates the 3-hydroxyl group of PtdIns-4, 5-bisphosphate (PIP2) to produce the second messenger PIP3. PIP3 recruits Pleckstrin homology (PH) domain-containing regulators to the inner plasma membrane. One main PI3K effector is protein kinase B (PKB/Akt) [16]. Besides Akt, PH-domain-containing cytoskeletal regulators sense PIP3 and mediate cortical actin dynamics at the so-called leading edge cytocortex. As such, the PH-like domain family B member 2 (*Phldb2,* hereafter referred to as LL5β) acts as a sensitive PIP3 effector during the establishment of planar cell polarity (PCP), lamellipodia formation, protrusion and subsequent chemotaxis [17]. LL5β orchestrates actin rearrangements through tethering actin cross-linkers of the filamin family to PIP3-rich plasma membranes [17-19]*.*

In this study, we identified that the PI3K regulatory subunit p55γ functions as a novel BMPRII-interacting protein. It acts in concert with p110α to mediate BMP2-induced PIP3 production and hence cortical actin rearrangements. We visualised that BMP2-induced PI3K activity produces PIP3 at the cytocortex, which subsequently recruits LL5β to orchestrate cortical actin crosslinking. Either knock-down of p55γ or LL5β or pharmacological inhibition of PI3K impaired BMP2-induced directional cell migration. Hence our study presents the first insights into the molecular activation and regulation mechanism by which BMP2 facilitates PI3K activity and the cytocortical signalling events leading to cortical actin reorganisation, PCP and chemotaxis. These molecular details are important to better understand BMP2-induced chemotaxis of mesenchymal progenitor cells during vertebrate development, tissue repair or disease.

## Results

### BMP2-induced PI3K signalling is required for chemotaxis

**Figure 1:**
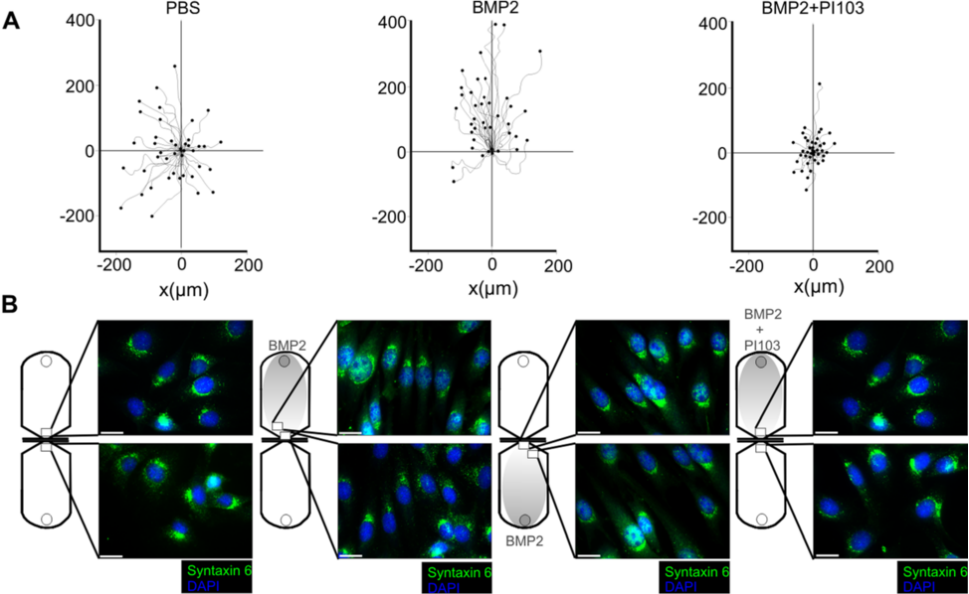
**BMP2 induces chemotaxis of multipotent mesenchymal C2C12 mouse myoblasts. (A)** Trajectories of multipotent mouse mesenchymal C2C12 cells migrating in a 2D chemotaxis chamber over period of 16 hours exposed to a linear BMP2 gradient compared to non-stimulated control or in the presence of the PI3K p110α selective inhibitor PI103 (8 nM). The gradient was produced by application of BMP2 to the upper reservoir. It was allowed to generate a linear concentration profile with a maximum concentration of approximately 10 nM reaching the cells on the edge of the observation area as described by the manufacturer. **(B)** Syntaxin 6 and DAPI stainings of C2C12 cells after BMP2-induced chemotaxis compared to non-stimulated control or PI103 [8 nM] pre-treatment. The location of the depicted cells within the chemotaxis chamber is indicated. Scale bar represents 20 μm. (2D) two dimensional; (BMP2) Bone Morphogenetic Protein 2; (PI3K) Phosphoinositide 3-kinase; (p110α) Class I PI3K catalytic subunit alpha; (DAPI) 4',6-diamidino-2-phenylindole.

To visualise BMP2-induced chemotaxis of multipotent mesenchymal progenitor cells, we used a 2D *in vitro* setup, which allowed the application of a linear BMP2 gradient and concomitant tracking of migrating C2C12 cells over time. Undifferentiated C2C12 myoblasts are multipotent and represent a common tool for investigating BMP signalling and its cellular functions. Non-stimulated cells displayed basal random migration, while application of a linear BMP2 gradient resulted in an overall gain in migratory directionality towards the source of BMP2 and a gain in migration distance. C2C12 cell chemotaxis was blocked upon pre-incubation with the PI3K p110α selective inhibitor PI103 (Figure 1A). Trans-Golgi staining of Syntaxin 6 in migrated C2C12 cells revealed PCP with the trans-Golgi aligned towards the leading edge, which was going with the direction of chemotaxis. By contrast, the Golgi were aligned randomly when cells were not stimulated or allowed to undergo BMP2-induced chemotaxis in the presence of PI103 (Figure 1B).

### PI3K regulatory subunit p55γ interacts with the long and short forms of BMPRII

**Figure 2:**
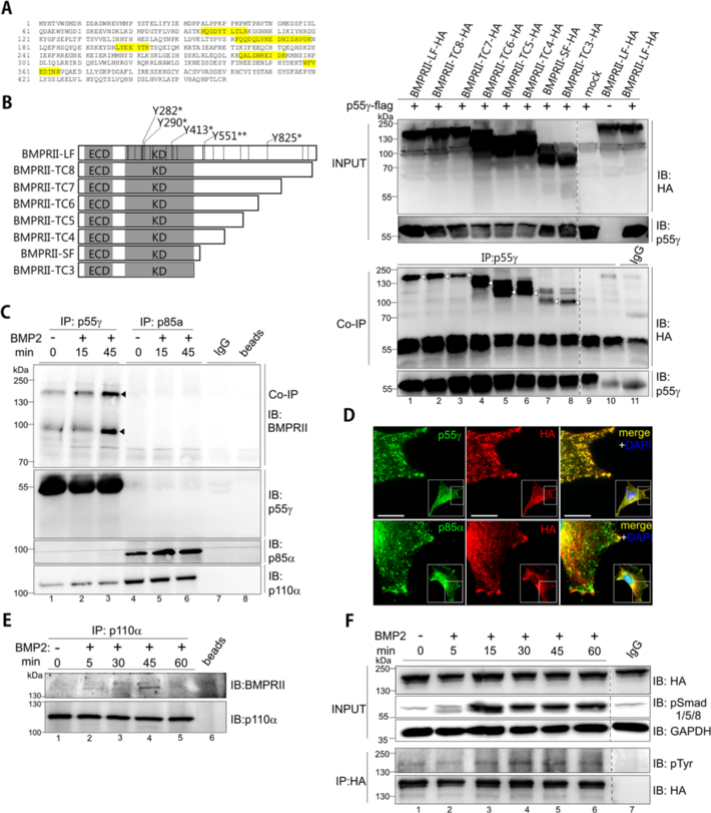
**PI3K regulatory subunit p55γ interacts with BMPRII and p110α. (A)** p55γ-specific peptides obtained after GST-BMPRII-pull-down from C2C12 lysates [20] are shown in yellow. **(B)** Scheme depicting BMPRII and truncations, including all tyrosines (black lines) and the ones serving as putative p55γ-SH2 domain binding sites (*). Tyrosines identified by alignment with known SH2 binding peptides (*) and oriented peptide library technique (**). Lines indicate localisation of 24 intracellular tyrosines in BMPRII. On the right, lanes 1 to 8 show input controls (top) and co-immunoprecipitation of BMPRII-LF, -SF or TC3-8 with p55γ from transfected HEK293T cells. The expected molecular weights of BMPRII and truncations are marked by white arrowheads. **(C)** Co-immunoprecipitation of endogenous BMPRII-LF and -SF (black arrowheads) with endogenous p55γ, but not p85α upon stimulation with 10 nM BMP2 for indicated time. The depicted western blots are representative of three independent experiments. **(D)** Immunocytochemical staining of C2C12 cells displaying co-localisation of endogenous p55γ, but not p85α, with overexpressed HA-tagged BMPRII-LF. Enlarged region of interest depicts co-localisation of p55γ but not p85α (green) with HA-tagged BMPRII-LF (red) at the cell periphery. Scale bar: 10 μm. **(E)** Co-immunoprecipitation from C2C12 lysates of endogenous BMPRII and endogenous p110α upon BMP2 stimulation for indicated time. **(F)** BMP2-induced tyrosine phosphorylation of BMPRII-LF. HEK293T cells transfected with HA-tagged BMPRII-LF were treated with 10 nM BMP2 for indicated time and subjected to immunoprecipitation with anti-HA antibody. Input controls of HA-tagged BMPRII-LF and BMP2-induced Smad 1/5/8 phosphorylation kinetics are shown (upper two panels). Fourth panel indicates BMPRII tyrosine phosphorylation through incubation of α-HA precipitates with pan anti-pTyr antibody. Dotted lines in F and B indicate deletion of non-relevant lanes. (See also Additional file 1: Figure S1C). (ECD) extracellular domain; (KD) kinase domain; (LF) long form; (SF) short form; (TC) truncation; (IgG) immunoglobulin G.

To address the molecular mechanism of BMP-induced directional cell migration, we followed some promising hits from a proteomics-based mass spectrometry screen designed to identify novel BMPRII interacting proteins [20]. Among those proteins not published earlier was PI3K regulatory subunit p55γ (Figure 2A, p55γ specific peptides in yellow) that co-immunoprecipitated with Glutathione S-transferase (GST)-tagged BMPRII short form (SF). BMPRII exists in mouse myoblast C2C12 cells in two splice variants, the BMPRII-long form (BMPRII-LF) and BMPRII-SF [21], with BMPRII-LF abundant in most other cell types. To first investigate the interaction site for p55γ in BMPRII, we performed co-immunoprecipitation studies in HEK293T cells upon overexpression of different BMPRII truncations (TCs) (Figure 2B) that lack parts of the C-terminal tail unique for BMPRII-LF. Upon p55γ precipitation we confirmed an interaction with wild-type (wt) BMPRII-LF and all BMPRII truncations (TC3 to TC8) as well as BMPRII-SF (Figure 2B). To validate the interaction of p55γ with both splice forms, we performed studies in C2C12 cells by pull-down of either endogenous p55γ or endogenous p85α (for antibody validation see Additional file 1: Figure S1A). We then probed for co-precipitated endogenous BMPRII by use of a BMPRII-specific antibody recognising an extracellular epitope (Figure 2C). As shown in lanes 1 to 3, endogenous p55γ, but not p85α (lanes 4 to 6), co-immunoprecipitated with BMPRII-LF and BMPRII-SF, with the receptor association to p55γ increasing over time during BMP2 treatment. Furthermore, we detected the class Ia catalytic subunit p110α in p55γ precipitates, suggesting that BMP2 activates PI3K heterodimers of p55γ and p110α (Figure 2C, lower panel lanes 1 to 3). Since co-immunoprecipitation in C2C12 cells confirmed a p55γ but not p85α interaction with BMPRII, we compared their respective co-localisation patterns in intact cells. For this, C2C12 cells were transiently transfected with Human influenza hemagglutinin (HA)-tagged BMPRII-LF and stained by use of antibodies binding to regulatory subunits and the HA-tag. Epifluorescence microscopy revealed strong co-localisation of p55γ, but only partial co-localisation of p85α, with BMPRII-LF within C2C12 cell protrusions (Figure 2D). Co-localisation was quantified defining a fixed region of interest. Mean Pearson’s coefficient of three sets of independent experiments revealed 0.933 ± 0.092 for co-localisation of p55γ and 0.741 ± 0.093 for p85α with BMPRII-LF (Additional file 1: Figure S1B). We then confirmed that p110α indeed specifically binds to BMPRII by precipitation of endogenous p110α which co-immunoprecipitated BMPRII in a BMP2-dependent manner (Figure 2E, lane 4). Together, these data demonstrate that p55γ specifically binds to BMPRII irrespective of the presence of the C-terminal tail and is part of a p110α-containing PI3K complex.

### BMPRII becomes tyrosine phosphorylated in a BMP2-dependent manner

Class Ia PI3Ks interact with activated growth factor receptors via pTyr motifs recognised by the SH2 domains of the regulatory subunit [22]. BMPRII is a serine/threonine kinase and its tyrosine phosphorylation has not been investigated to our knowledge. The cytosolic part of BMPRII-LF contains 24 tyrosines; the majority of tyrosines are located within the kinase domain, a few in the C-terminal tail and none in the juxtamembrane region preceding the kinase domain (Figure 2B). An *in silico* alignment of the BMPRII cytosolic domain with known SH2 domain-binding peptides (Figure 2B, marked with *) [15] and analysis using ScanSite oriented peptide library technique (marked with **) [23] identified five potential tyrosines that could act as SH2 domain docking sites (black lines indicate locations of all other BMPRII tyrosines in cytosolic domains). To first analyse BMP2-dependent tyrosine phosphorylation of BMPRII, we transfected HEK293T cells with HA-tagged BMPRII-LF, followed by immunoprecipitation using anti-HA antibody. BMPRII tyrosine phosphorylation was investigated using an anti-pTyr antibody. We found basal Tyr phosphorylation of BMPRII-LF in starved cells (Figure 2F, lower panel, lane 1), which increased upon 15 to 60 minutes stimulation with BMP2 (lanes 3 to 6). This kinetic profile resembles Smad1/5/8 phosphorylation by activated receptor complexes (Figure 2F, upper panel). A BMP2-dependent Tyr phosphorylation of endogenous BMPRII was also confirmed using C2C12 cells upon pull-down of endogenous BMPRII after 60 minutes’ BMP2 stimulation compared to non-stimulated control (Additional file 1: Figure S1C,D,E). A vice versa approach by performing a pTyr pull-down upon BMP2 stimulation on BMPRII-LF-HA transfected HEK293T cells and subsequent blotting using anti- HA antibody also confirmed the tyrosine phosphorylation of BMPRII (Additional file 1: Figure S1D). The pTyr specificity of the antibody was proven by sodium orthovanadate treatment of cells and additionally by dephosphorylation using Antarctic phosphatase treatment of the membrane after western blotting with pTyr antibody (Additional file 1: Figure S1E). To identify particular phosphorylated tyrosine residues on BMPRII, respective mass spectrometry approaches have to be performed in the future. Together, these results confirm that BMPRII is tyrosine phosphorylated in a BMP2-dependent manner and provides the required features to associate with p55γ.

### BMPRII-kinase activity is dispensable but the presence of BMPRI enhances BMPRII-p55γ interaction

**Figure 3:**
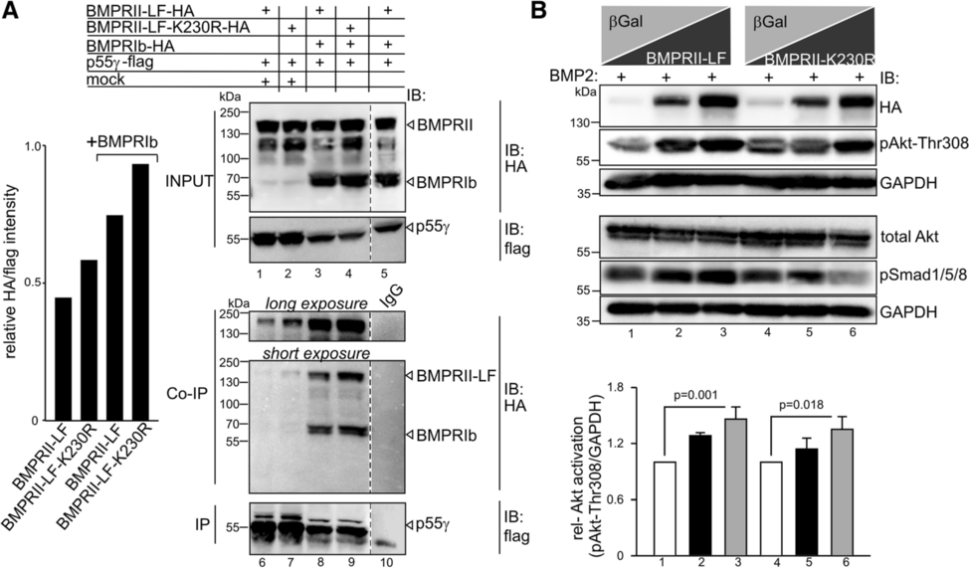
**BMPRII-kinase activity is dispensable but BMPRI enhances the p55γ interaction with BMPRII. (A)** Co-immunoprecipitation of HA-tagged BMPRII-LF or kinase-dead BMPRII-K230R-HA with flag-tagged p55γ in the presence or absence of BMPRIb-HA from transiently transfected HEK293T cells. Left panel depicts quantification of co-immunoprecipitated BMPRII relative to the amount of flag-tag mediated precipitation of p55γ. Upper panel shows input controls for BMPRII-HA, BMPRIb-HA and p55γ-flag. Lower panel depicts co-immunoprecipitation of BMPRII-HA with blots taken with long (upper lanes) and short (lower lanes) exposure times. White arrowheads indicate the migration heights of BMPRII, BMPRIb and p55γ. Dotted lines indicate deletion of non-relevant lanes from the same blot. **(B)** Activation of PI3K signalling by transient expression of BMPRII and BMPRII-K230R. Upper panel shows the effect of increasing amounts of BMPRII-LF or BMPRII-K230R transfected cells against β-galactosidase (β-gal) on the activation of phospho-Akt Thr308 in HEK293T cells. Experiments were carried out in the presence of 10 nM BMP2, which was added 60 minutes prior to cell lysis. Note the dominant negative effect of BMPRII-LF-K230R on pSmad1/5/8 but not phospho-Akt Thr308. Lower panel shows quantification of phospho-Akt Thr308 intensities relative to GAPDH. Error bars represent standard deviation from three independent experiments. *P*-values from one-way analysis of variance with post-hoc Bonferroni-test are indicated. (See also Additional file 2: Figure S2.)

BMP receptor complexes comprising BMPRI and BMPRII oligomerise by different modes with the BMP induced signalling complex (BISC) to induce non-Smad signalling [24,25]. BISCs are formed through a BMP2-induced recruitment of BMPRII to ligand-bound BMPRI and this is required for the induction of non-Smad pathways [25,26]. To investigate the contribution of BMPRII kinase activity in the BMPRII-p55γ complex, we first investigated the binding properties of flag-tagged p55γ to HA-tagged wt BMPRII-LF compared to binding to a kinase-dead mutant (BMPRII-LF-K230R) [27]. Upon overexpression in HEK293T cells and precipitation of p55γ, we detected both wt BMPRII-LF and BMPRII-LF-K230R in p55γ precipitates (Figure 3A, lanes 1 and 2). Intriguingly, we found the interaction of p55γ with wt BMPRII-LF and BMPRII-LF-K230R was further facilitated by concomitant overexpression of BMPRIb (Figure 3A, lanes 3 and 4). By contrast, BMPRIb alone or the corresponding BMPRI kinase-dead mutant (BMPRIb-K231R) did not co-immunoprecipitate with p55γ (Additional file 2: Figure S2). These data prove that the kinase activity of BMPRII is dispensable for association with p55γ, whereas the availability of BMPRI critically influences the interaction of p55γ to BMPRII. To elucidate further whether BMPRII-LF and BMPRII-LF-K230R are equally potent in activating signalling by PI3K, we expressed increasing amounts of each receptor in HEK293T cells followed by detection of phospho-Akt threonine 308 (Thr308). In the presence of BMP2, both wt BMPRII-LF and BMPRII-LF-K230R significantly promoted Akt phosphorylation at Thr308 as the amount of DNA transfected was increased (Figure 3B). As expected, expression of BMPRII-LF-K230R resulted in a dominant negative effect on the BMP2-induced Smad signalling, seen by a decreased Smad1/5/8 phosphorylation (Figure 3B, lanes 4 to 6).

### BMP2-induced PI3K signalling is specifically mediated via p55γ

**Figure 4:**
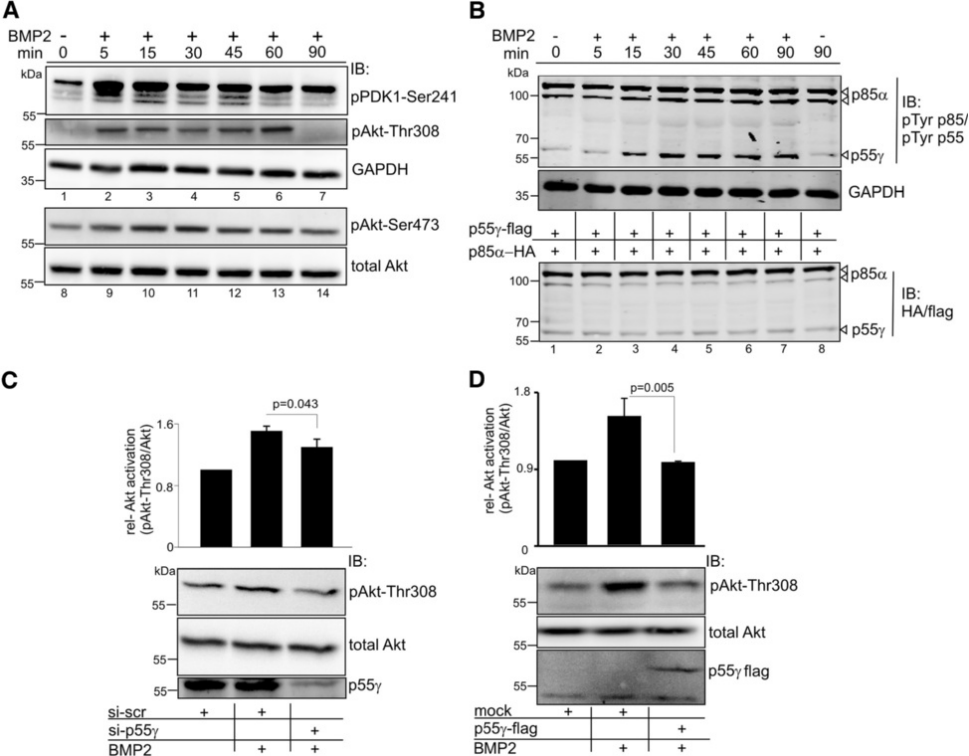
**BMP2-induced PI3K signalling is specifically mediated via p55γ. (A)** Phospho-kinetics of PI3K effector proteins in C2C12 upon stimulation with 10 nM BMP2 for the indicated time. Phosphorylation of PDK1 (Ser241), Akt (Thr308) and Akt (Ser473) was analysed. **(B)** BMP2-dependent tyrosine phosphorylation at the inter-SH2 domain of PI3K regulatory subunits p55γ and p85α. HEK293T cells were transfected with equal amounts of p55γ-flag and p85α-HA and stimulated with 10 nM BMP2 for the indicated time. Upper panel depicts BMP2-dependent phosphorylation of conserved Tyr458 of p85α (see arrowheads, double band at approximately 100 kDa) corresponding to Tyr199 of p55γ (see arrowhead at approximately 55 kDa). The detected signals migrated accordingly to the signals of p85α-HA and p55γ-flag in the expression control (arrowheads, lower panel). **(C)** Knock-down of p55γ reduces BMP2-induced Akt-Thr308 phosphorylation of C2C12 cells upon 60 minutes’ stimulation with BMP2. The relative phospho-Akt-Thr308 to GAPDH levels were determined. **(D)** Overexpression of p55γ-flag in C2C12 cells reduces BMP2-induced Akt-Thr308 phosphorylation upon 60 minutes’ stimulation with BMP2. The relative phospho-Akt-Thr308 to GAPDH levels were determined. For experiments C and D: error bars represent standard deviation from three independent experiments. *P*-values from one-way analysis of variance with post-hoc Bonferroni-test are indicated. (See also Additional file 4: Figure S4.)

We next characterised the dynamics of BMP2-induced PI3K signalling in C2C12 cells, focusing on main PI3K-PIP3 effectors to show definitively that p55γ is required for PI3K signalling. We detected immediate (after 5 minutes; Figure 4A, lanes 2 and 3) phosphorylation of 3-phosphoinositide-dependent kinase-1 (PDK1), coinciding with phosphorylation of Akt at Thr308; phosphorylation of Akt at Ser473 was detected after 15 minutes (Figure 4A, lanes 10 to 13). Phosphorylation of several tyrosines in PI3K regulatory subunits by PI3K agonists has been previously demonstrated and phosphorylation of the inter-SH2 domain (iSH2) was suggested to mediate receptor specificity [28] and p110 catalytic activity [29]. We probed for phosphorylation of PI3K regulatory subunit iSH2 using a pTyr-specific antibody, which detects a conserved Tyr within the iSH2 domain. This antibody has been previously used to probe for PI3K activation in response to Src [30]. To discriminate between BMP2 effects on iSH2 Tyr-phosphorylation of p55γ (Tyr199) and p85α (Tyr458), equal amounts of flag-tagged p55γ and HA-tagged p85α were expressed in HEK293T cells. BMP2 stimulation resulted in a time-dependent phosphorylation of p55γ-Tyr199 after 15 minutes, whereas p85-phosphorylation appeared less affected (Figure 4B). Subsequently, we investigated whether BMP2-induced PI3K signalling is p55γ-dependent. For this, we performed siRNA-mediated knock-down of endogenous p55γ (knock-down control, see Additional file 3: Figure S3). As expected, siRNA-mediated knock-down of p55γ significantly impaired BMP2-induced Akt phosphorylation at Thr308 compared to a scrambled siRNA control (Figure 4C). In addition, we investigated the effect of p55γ overexpression on BMP2-induced Akt phosphorylation. We found that p55γ overexpression exerts a dominant negative effect on BMP2-induced Akt phosphorylation, a phenomenon that has been previously reported to underlie an unbalanced ratio between the regulatory and catalytic subunits [31] (Figure 4D). Taken together, these results demonstrate that p55γ specifically links BMP2 with the activation of PI3K signalling.

### BMP2-induced PIP3 production is dependent on p55γ

**Figure 5:**
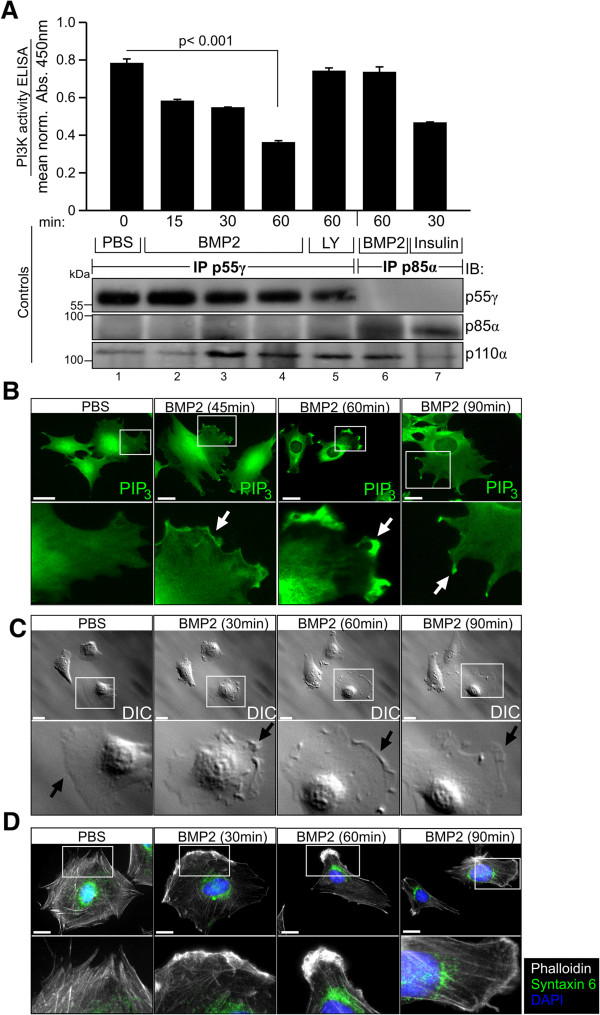
**BMP2-induced PIP3 production is p55γ-dependent and localises to cortical actin during lamellipodia formation. (A)** BMP2-dependent PIP3 production as detected by PI3K activity ELISA (upper panel). C2C12 cells were stimulated with respective ligands and inhibitors for the indicated times and lysates were subjected to pull-down of endogenous p55γ or p85α as shown. Precipitates were subjected to an *in vitro* kinase reaction and competitive ELISA was used to detect the amount of PIP3 produced by BMP2-induced PI3K activity. Low absorbance at 450 nm indicates high levels of PIP3. To prove the presence of catalytic p110α in PI3K regulatory subunit pull-down, bead lysates of all three assays were pooled and subjected to detection of p55γ, p85α and p110α protein respectively (lower panel). Error bars represent standard deviation from three independent experiments. **(B)** Immunocytochemical detection of PIP3 in BMP2 (10 nM) stimulated C2C12 cells by use of PIP3-specific antibody. The cortical region of PIP3 accumulation is indicated by the white arrow. **(C)** Differential interference contrast (DIC) microscopy of membrane ruffles at dorsal (white arrow) regions of the leading edge of C2C12 cells stimulated with 10 nM BMP2. In B and C, the lower boxes depict magnifications of the regions of interest indicated by white squares (upper boxes). Scale bars represent 20 μm. **(D)** Phalloidin and Synatxin 6 staining indicating trans-Golgi position facing the cortical actin-rich leading edge of C2C12 cells stimulated with 10 nM BMP2 for the indicated time. Scale bars represent 10 μm. (See also Additional file 5: Figure S5).

We then analysed whether BMP2-induced PIP3 production requires p55γ by performing a PI3K activity assay. For this, C2C12 cells were stimulated with BMP2 following pull-down of p55γ or p85α. Subsequently, we analysed *in vitro* lipid kinase activity of precipitated complexes using a competitive ELISA system (Figure 5A). Precipitates of p55γ revealed increased PIP3 production after BMP2 stimulation for 15 minutes (lane 2; decreased absorbance at 450 nm), which further increased at the 60-minute time-point (lanes 2 to 4). By contrast, pre-treatment with the PI3K inhibitor LY-294002 or pull-down of p85α gained PIP3 levels comparable to levels in non-stimulated p55γ precipitates (Figure 5A, lanes 5 and 6). The pull-down of p85α only resulted in elevated PIP3 levels when cells were stimulated with insulin (100 nM). This further underlines the role of p85α in other pathways, but not BMP signalling (Figure 5A, lane 7). Pull-down controls for both regulatory subunits and the co-immunoprecipitated p110α are shown (Figure 5A, lower panel). The potency of small molecule inhibitors in interfering with BMP2-induced PI3K signalling was tested by the application of Wortmannin (25nM) (Additional file 4 Figure S4) and LY-294002 (10 μM), the class Ia selective PI3K inhibitor PI103 (8 nM) or the BMPRI-kinase specific inhibitor LDN-193189 (0.5 μM) (Additional file 5: Figure S5A)*.*

### PIP3 and PIP3 effectors localise to BMP2-induced cortical actin-rich lamellipodia

The p55γ-dependent production of PIP3 led us to the hypothesis that BMP2-induced cytoskeletal rearrangements utilise membrane-anchored PIP3 to target actin-reorganising proteins to the cytocortex. Staining with PIP3-specific antibody revealed increased PIP3 accumulation within dorsal ruffles and lamellipodial protrusions upon BMP2 stimulation (Figure 5B). Consistent with this, pre-incubation with PI103 blocked the BMP2-dependent translocation of the GFP-tagged PH-domain of Akt (Additional file 5: Figure S5B) and the localisation of phospho-Akt and phospho-PDK1 to BMP2-induced actin-rich lamellipodia (Additional file 5: Figure S5C). To characterise the dynamics of PIP3-enriched lamellipodia, we performed live-cell imaging combined with differential interference contrast microscopy. Application of BMP2 to living cells induced dynamic cytoskeletal rearrangements and dorsal ruffling (0 to 30 minutes) followed by a sustained lamellipodia protrusion phase (30 to 90 minutes) (Figure 5C, Additional file 6). This response was accompanied by an overall change in leading edge directionality (Figure 5C, black arrows). Subsequent actin staining uncovered BMP2-induced lamellipodia enriched in cortical actin. Concomitant staining using an anti-Syntaxin 6 antibody indicated that the Golgi apparatus realigned upon BMP2 stimulation to face the cells leading edge (Figure 5D). We also found that in C2C12 cells, endogenous BMPRII-LF localises to BMP2-induced dorsal ruffles independent of new protein synthesis as proven by cyclohexamide treatment but also independent of canonical Smad signalling using LDN193189 (Additional file 5: Figure S5D).

### The PIP3-binding protein LL5β localises to BMP2-induced cortical actin-rich lamellipodia

**Figure 6:**
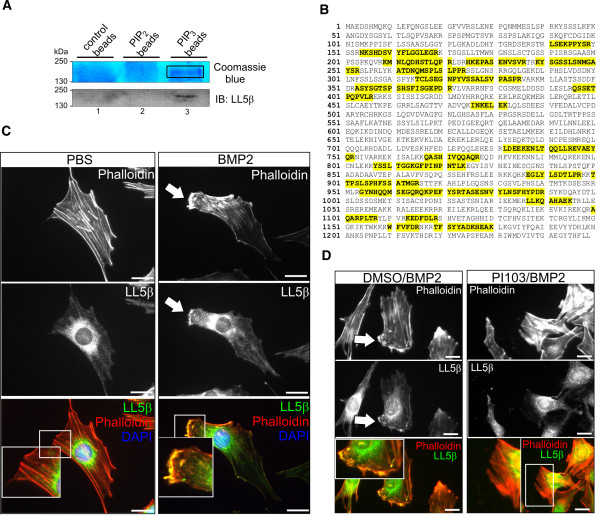
**The PIP3-binding protein LL5β localises to BMP2-induced cortical actin-rich lamellipodia. (A)** Upper panel shows colloidal Coomassie Blue staining of protein precipitates gained by precipitation of PIP2-, PIP3-coated and control beads from C2C12 cell lysates. Lower panel shows LL5β detection (approximately 160 kDa) by western blot. LL5β binds to PIP3 (lane 3) but not PIP2 or control beads (lanes 1 and 2). **(B)** LL5β-specific peptides (marked in yellow) as identified by mass spectrometry upon precipitation of PIP3-coated beads from C2C12 cell lysates. **(C)** Immunocytochemical stainings of endogenous LL5β and actin in C2C12 cells upon 45 minutes’ stimulation with 10 nM BMP2. Arrow indicates co-localisation of LL5β with cortical actin in BMP2-induced cell protrusions at the C2C12 cell leading edge (magnified region of interest). **(D)** PI103 pre-treatment blocks BMP2-induced co-localisation of LL5β with cortical actin. C2C12 cells were stimulated with 10 nM BMP2 for 45 minutes in the presence of DMSO or 8 nM PI103 respectively. The magnified region of interest depicts the co-localisation of LL5β with cortical actin. Scale bars represent 20 μm.

Regulators of cortical actin that transduce BMP2 signals in a PIP3-dependent manner are largely unknown. To identify putative BMP2-dependent and PIP3-sensitive cytoskeletal regulators, we performed pull-downs in C2C12 cell lysates using PIP3-coated beads following mass spectrometry. We showed that the 160 kDa protein LL5β bound specifically to PIP3 (Figure 6A), whereas LL5β was absent from PIP2 precipitates and control beads (Figure 6A, specific peptides for LL5β shown in Figure 6B). LL5β is recruited by PIP3 to the cytocortex in complex with filamins, which are major filamentous actin (F-actin) cross-linkers [19]. To prove that LL5β is involved in BMP2-dependent cortical actin rearrangements, we first analysed its sub-cellular localisation. In resting C2C12 cells, LL5β localised to a cytosolic compartment surrounding the nucleus with a sparse distribution towards the cell cortex (Figure 6C). Upon BMP2 stimulation, LL5β translocated to the leading edge cytocortex where it co-localised with cortical actin (Figure 6C). Pre-incubation with PI103 resulted in loss of BMP2-induced cortical actin filaments and LL5β remained at cytosolic compartments (Figure 6D). Collectively, these data indicate that the PH-domain protein LL5β is involved in BMP2-induced actin reorganisation at the leading edge cytocortex through recruitment by PIP3.

### PI3K p55γ/p110α and LL5β are required for BMP2-induced migration and chemotaxis

**Figure 7:**
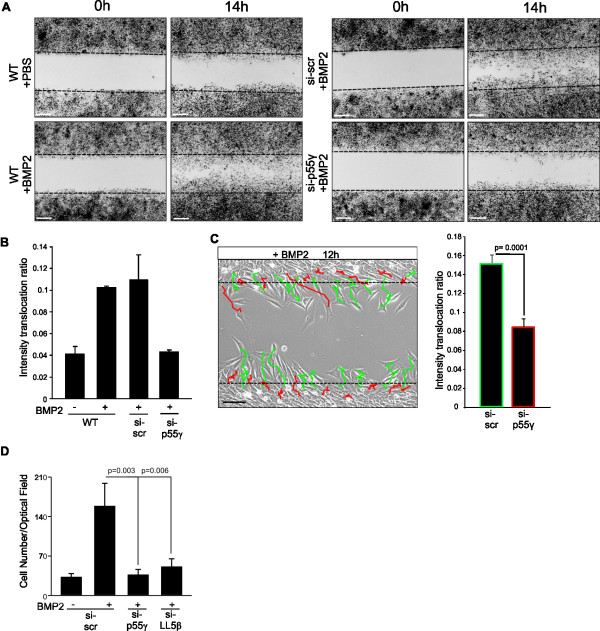
**BMP2-induced PI3K signalling is important for directional cell migration. (A)** C2C12 cell wound closure upon stimulation with 10 nM BMP2 for 14 hours compared to unstimulated control. Lipophilic carbocyanine-dye labelled cells (DiO) are presented in black pseudo-colour. Scale bar represents 200 μm. Left panel, effect of p55γ knock-down (si-p55γ) on C2C12 cell wound closure compared to scrambled transfected (si-scr) control. **(B)** Bar diagram depicting the intensity translocation values for three independent biological replicates for experiments shown in A using a selective mask filter (for detailed description see Methods and Additional file 7). Error bars represent standard deviation from three independent experiments. *P*-values from one-way analysis of variance with post-hoc Bonferroni-test are indicated. **(C)** Trajectories visualising the migration of p55γ knock-down (red) and scrambled control (green) C2C12 cells during BMP2-induced wound closure over a period of 12 hours. Scale bar represents 100 μm. Left panel, equal amounts of si-p55γ (red) and si-scr (green) transfected cells were labelled with DiI or DiO respectively prior to mixing and seeding. Bar diagram (right panel) summarises the intensity translocation ratios of p55γ knock-down (red) compared to scrambled control (green) C2C12 cells of 19 replicates analysed with *P* <0.005 considered statistically significant. **(D)** Transwell assay of C2C12 cells, transfected with either si-p55γ or si-LL5β compared to si-scr-transfected C2C12 cells. Cells migrated through an 8 μm porous filter upon stimulation with 10 nM BMP2 for 6 hours in the presence of 0.2% fetal calf serum. The number of cells migrated through the porous filter was counted. Bar diagram represents cell number counts per optical field. Error bars represent standard deviation from three independent experiments. *P*-values from one-way analysis of variance with post-hoc Bonferroni test are indicated.

**Figure 8:**
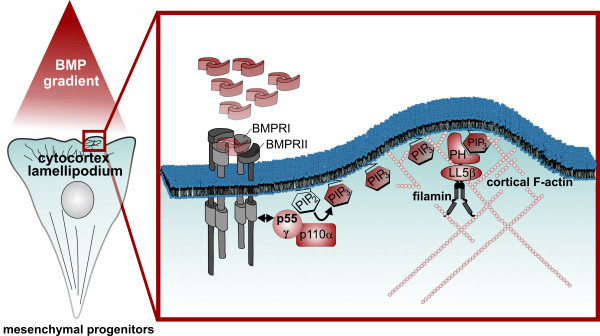
**Scheme depicting BMP2-induced PI3K signalling via p55γ/p110α and cortical actin rearrangements via PIP3-LL5β filamins in mesenchymal progenitor cells.** Gradients of BMP2 activate the BMP receptor complex and facilitate the association of BMPRII to regulatory subunit p55γ coupled to class Ia catalytic subunit p110α. The recruitment and activation of PI3K generates the membrane-bound second messenger PIP3 at the leading edge of cells that are about to establish PCP. PIP3 recruits the PH-domain protein LL5β. LL5β co-recruits and tethers the actin crosslinker filamin to the leading edge, where it promotes actin polymerisation, crosslinking and subsequent initiation of lamellipodia formation, extension and protrusion. BMP2, bone morphogenetic protein 2; BMPRI/II, bone morphogenetic protein receptor type I/II; pSmad1, phospho-Smad1; p55γ, PI3K regulatory subunit p55 gamma; p110α, p110 catalytic subunit p110 alpha; PIP2, phosphatidylinositol (4,5)-bisphosphate; PIP3, phosphatidylinositol (3,4,5)-triphosphate; LL5β, pleckstrin homology-like domain family B member 2; PH, pleckstrin homology domain; F-actin, filamentous actin.

To confirm the cellular function of our molecular findings, we investigated whether BMP2 promotes cell migration, in particular wound closure, which requires a distinct cell polarity. We found that BMP2 was able to increase the speed of C2C12 wound closure relative to a non-stimulated control within 14 hours (Figure 7A). Additionally, knock-down of p55γ impaired BMP2-induced wound closure compared to control transfected cells. Intriguingly, we found that knock-down of p55γ significantly reduced the ability of cells to efficiently enter the wound in a BMP2-dependent fashion (for quantification see Figure 7B). We also investigated the relative migration of p55γ knock-down cells (red) compared to scrambled transfected cells (green) by seeding a ‘salt and pepper’ mix within the same wound. p55γ knock-down cells displayed considerably impaired polarity and thus reduced ability to efficiently enter the wound, instead displaying short trajectories (DiI: fluorescent lipophilic cationic indocarbocyanine dye/red) compared to control cells (DiO: fluorescent lipophilic cationic indocarbocyanine dye/green; Figure 7C). Next, we performed a transwell assay to analyse whether the effect of BMP2-induced migration becomes more prominent when cells are exposed to a ligand gradient. We found that BMP2 induced transmigration of C2C12 cells, whereas knock-down of p55γ or LL5β significantly impaired this response (Figure 7D).Collectively, our results demonstrate that the BMPRII-p55γ interaction is necessary for BMP2-induced class Ia PI3K activation via the BMPRII-p55γ interaction and PIP3 production via p110α activity at the leading edge cytocortex. Moreover, we showed that the BMP2-induced activation of PI3K is critically involved in actin reorganisation and lamellipodia formation due to the production of PIP3 and LL5β recruitment. With LL5β, we found an important PIP3 effector and actin regulator through its well-described role in tethering filamins to the cytocortex. p55γ, p110α and LL5β, therefore, critically influence BMP2-induced chemotaxis with p55γ being a novel and specific BMPRII-interacting protein required for chemotactic mesenchymal progenitor cell responses to BMP2 (Figure 8).

## Discussion

Since the initial discovery that BMPs act as chemotactic guidance cues [32], the molecular mechanism of how BMPs initiate cell migration and chemotaxis has remained poorly understood. However, an important role for BMP-induced cell migration has been demonstrated in several excellent developmental [2,3,33], repair and disease studies [9,34]. Here, we aimed to close a gap in the mechanistic molecular understanding of how BMPs in general activate PI3K signalling in progenitor cells at the molecular level and how this influences actin reorganisation at the cytocortex and, hence, lamellipodia formation. We uncovered major and crucial aspects of the molecular mechanism by which BMP2 initiates and extends PI3K-signalling at the plasma membrane, visualised and localised BMP2-induced PIP3 for the first time in intact cells, and confirmed the requirement of p55γ and LL5β for BMP2-induced migration and chemotaxis of mesenchymal progenitor cells.

### The role of the BMP receptor complex in activating PI3K signalling

Here, we describe the specific association of the class Ia PI3K regulatory subunit p55γ with BMPRII for the first time. This interaction is enhanced by either BMP2 stimulation or the presence of BMPRI whereas the kinase activity of BMPRII seems dispensable. This observation may reflect the same mechanism by which BMPRII is incorporated into BISCs upon stimulation with BMP2 [24], where the high affinity receptor for BMP2 (BMPRI) recruits BMPRII into the complex upon BMP2 binding. Moreover, we showed previously that BISC-mediated signalling and BMPRII recruitment towards BMPRI is required for non-Smad signalling [25,26]. We therefore speculate that the BMPRI kinase is required for PI3K activation whereas BMPRII acts as a scaffolding hub to provide PI3K for BMPRI-dependent activation mechanisms that have not yet been defined. This hypothesis is underlined by our previous findings of reduced BMP2-induced Akt phosphorylation upon pharmacological inhibition of BMPRI kinase activity [35] (see also Additional file 5: Figure S5A). BMPRI activity seems crucial in mediating the association of p55γ with BMPRII and, thus, PI3K activity. Research on the related TGF-β pathway identified that the high affinity TGF-β receptor type II associated constitutively with p85α, whereas the low affinity TGF-β type I receptor only associated with p85α in a ligand-dependent manner [36]. However, it should be considered that BMPRI is the high affinity and BMPRII the low affinity receptor for BMP2. This would therefore represent a mirror-image scenario of PI3K regulatory subunit interaction in BMP versus TGF-β receptors. Tyrosine phosphorylation of BMPRII is essential for an association with class Ia PI3K p55γ. Despite its classification as a tyrosine-like kinase [37], a BMPRII dual kinase activity *in vivo* is still speculative and needs to be proven. Our experiments have shown that BMP2 stimulation rapidly induces BMPRII tyrosine phosphorylation *in vitro*, comparable to the kinetics of Smad1/5/8 phosphorylation via a yet unknown mechanism. Moreover, we identified BMPRII tyrosine residues that could act as direct putative SH2 domain docking sites. Since the interaction site for p55γ could be mapped to the BMPRII kinase, we speculate that pTyr motifs in the BMPRII kinase domain are required for its interaction. However, with the techniques applied here, we cannot comment on potential intermediate adaptor proteins or additional tyrosine kinases facilitating p55γ interaction and BMP2-dependent BMPRII tyrosine phosphorylation respectively. Along the same line, studies on the related activin pathway have already suggested the involvement of additional adaptor proteins that facilitate the interaction of PI3K regulatory subunits to the activin receptor ActRII [38]. The tyrosine kinases TrkC [39] and Src [40] also interact with BMPRII and could thus facilitate or mediate its tyrosine phosphorylation at sites required for the interaction to p55γ. Taken together, the BMP2-dependent tyrosine phosphorylation of BMPRII provides the required features for interaction with p55γ, but further research will be required to unravel the contribution of yet unknown tyrosine kinases and adaptor proteins that may be involved in this interaction.

### Exclusive role for p55γ in BMP2-induced PI3K signalling

To date, data regarding unique functions of p55γ are poor, mainly because it is speculated that the five different PI3K regulatory subunits have redundant functions and may compensate for each other. The data presented here show that p55γ provides specific functions during BMP2-induced PI3K signalling. This is underlined by its exclusive association with BMPRII, its BMP2-dependent phosphorylation in the iSH2 domain, and the effects on Akt phosphorylation and cell migration when knock-down of p55γ was performed. We have confirmed that, besides p55γ, all other class Ia regulatory subunits, namely p85α (including splice isoforms p55α and p50α) and p85β, are detectable at the mRNA level in undifferentiated multipotent C2C12 cells (data not shown). A prominent role for PI3K regulatory subunits during cytoskeletal rearrangements has already been described, especially in the context of actin reorganisation [41]. Interestingly, some studies have proposed that PI3K regulatory subunits provide non-redundant signalling functions dependent on their sub-cellular localisation within a cell [42,43]. This is in line with our data, showing that p55γ, but not p85α, interacts and co-localises with BMPRII, predominantly at the cell periphery. It still remains unclear how BMPRII selectivity for p55γ over p85α is achieved. The p55γ high-resolution crystal structure has not been determined and the SH2 and iSH2 domains of human p85α and p55γ share about 81.1% sequence identity. Based on the data presented here, we now propose two possible mechanisms by which BMPRII selectivity for p55γ could occur. First, our research revealed BMP2-dependent phosphorylation of the conserved Tyr199 within iSH2 of p55γ, but not p85α. Phosphorylation of p55γ iSH2 could induce structural changes, favouring an association of p55γ with BMPRII over that of the p85α SH2 domain. Second, the N-terminal 34 residues of p55γ bind to tubulin [42]. Because the p55γ N-terminal sequence is unique and not present in p85α, it was proposed that this interaction specifically recruits p55γ to the cell periphery [42]. During onset of cortical actin rearrangements, microtubule plus ends penetrate the leading edge cytocortex together with actin nucleating factors [44]. The binding of p55γ to microtubules, especially at the very tip, could thus provide a sub-cellular pool of p55γ for signalling involved in cortical actin-driven lamellipodia formation.

Besides specific functions of the class Ia PI3K regulatory subunits, class I catalytic subunits also attract increasing attention to provide non-redundant signalling functions [14]. The catalytic subunit p110α has been implicated in BMP2-induced PI3K signalling and cell migration by others using a pharmacological targeting approach [10]. In line with those observations, we found that p110α is in complex with p55γ and BMPRII. Moreover, this complex produced PIP3 in a BMP2-dependent fashion. Thus, we propose that BMP2-induced PI3K signalling is transduced specifically by the p55γ/p110α class Ia PI3K complex. This could be of particular importance for cancer therapy because activating mutations in p110α are frequently found in human cancers, and p55γ is differentially up-regulated in several tumours, which is sufficient to stimulate tumour angiogenesis [45]. This, together with the crucial role of BMP2 in oncogenic transformation and tumour angiogenesis [46-48], suggests that the p55γ/p110α complex positively regulates BMP2-induced motility, chemotaxis, and invasion of endothelial and cancer cells [9,49,50]. Whether the PI3K p55γ/p110α dimer indeed represents an attractive molecular target to interfere with BMP2-related cancers will require intense investigations in future.

### BMP2-induced PIP3 acts as a cellular compass at the leading edge and recruits LL5β

Numerous cellular activities have been reported to depend on BMP2-induced PI3K signalling [9-11,51-56]. Most previous studies focused on the role of PI3K-induced Akt activity with Akt being the major PI3K effector. In the present study, we investigated the role and function of PIP3 beyond Akt activation and focused on PIP3 localisation and recruitment of cytoskeletal regulators. We visualised BMP2-dependent PIP3 production in a spatiotemporal manner to gain further insight into its function. We found PIP3 became quickly enriched in BMP2-induced lamellipodia at the cytocortex, especially in cells that displayed strong PCP, suggesting that PIP3 acts as a cellular compass at the leading edge of migrating cells. PIP3 recruits PH-domain-containing proteins that facilitate rearrangements of the actin cytoskeleton [57]. With this knowledge, we aimed to identify PH-domain proteins that link BMP2-induced PIP3 to actin regulators. The BMP2-induced lamellipodia are tightly cross-linked F-actin networks located at the cytocortex of the leading edge. During maturation and protrusion, these actin-rich lamellipodia form broad lamella that allow for the formation of new adhesion sites [58]. In agreement with our observations, we identified a specific interaction of PH-domain protein LL5β with PIP3. LL5β acts as a highly sensitive PIP3 effector during epidermal growth factor-induced chemotaxis and lamellipodia formation [17]. It regulates the actin cytoskeleton through interaction with and co-recruitment of filamin C [19] and filamin A [17]. Filamins orchestrate cortical actin into three-dimensional structures by cross-linking of F-actin filaments [59]. Interestingly, besides tethering filamins, LL5β also tethers Cytoplasmic linker associated proteins (CLASPs) to the leading edge [17,18]. CLASPs attach microtubule tips to the cell cortex, which is important for microtubule stabilisation and thus PCP. Therefore, our findings provide evidence that LL5β acts as a BMP2-dependent multifunctional PIP3-sensing scaffold that eventually also orchestrates microtubule stabilisation at the cytocortex and thus links BMP2-dependent actin rearrangements to microtubule stabilisation.

### p55γ and LL5β are required for BMP2-induced migration and chemotaxis

The potency of BMP2 in stimulating migration of cells with mesenchymal origin is well known. Here, we raised the question of whether our findings contribute in particular to BMP2-induced cortical actin rearrangements, PCP and chemotaxis. We demonstrated that loss of p55γ prevents cells from efficient PCP establishment during wound healing and that knock-down of p55γ or LL5β resulted in impaired BMP2-induced chemotaxis. We therefore conclude that the pro-migratory effects of BMP2 are driven by PI3K signalling leading to PIP3-dependent cytoskeletal actin rearrangements, and result mainly in directional migration explained by the ‘compass’ function of PIP3.

## Conclusions

Our molecular findings provide a basis for explaining the important mechanism of BMP2-induced cortical actin rearrangements and chemotaxis, which we have graphically summarised (Figure 8). The novel *in vitro* data presented here close gaps in our current understanding of how BMP2 gradients influence the cellular cytoskeleton and hence mesenchymal progenitor cell chemotaxis*.* Interestingly, PIP3 production increases the efficacy of cells in detecting and processing shallow chemokine gradients [60]. This suggests that the molecular mechanism identified here is important for mesenchymal progenitor cells that respond to BMP2 gradients *in vivo* where they might originate from distant locations. To visualise this *in vivo* in the context of our novel molecular findings will be the future goal and a translation of this knowledge towards the fields of developmental biology and regenerative medicine is expected.

## Methods

### Chemicals, recombinant growth factors and inhibitors

All chemicals were purchased from Sigma Aldrich unless stated otherwise. Recombinant human BMP2 was kindly provided by Walter Sebald (University of Würzburg, Würzburg, Germany). The inhibitor LDN-193189 was a kind gift from Paul Yu (Harvard Medical School, Boston, MA, USA) and described elsewhere [61]. LY294002 was purchased from Cell Signaling Technology (Cell Signaling Technology Inc., Danvers, MA, USA) and PI103 was purchased from Echelon Bioscience (Echelon Bioscience Inc., Salt Lake City, USA).

### Antibodies

Phospho-specific antibodies, as well as protein- and tag-specific antibodies, were used and applied as recommended by the manufacturer. A detailed list of all antibodies used in this study is provided in Additional file 7.

### Cell culture

C2C12 cells and HEK293T cells (both from American Type Culture Collection) were cultivated in Dulbecco’s modified Eagle’s Medium (DMEM) (Biochrom GmbH, Berlin, Germany) supplemented with 10% (v/v) foetal calf serum and 100 U/ml penicillin/streptomycin. To maintain highest plasticity, C2C12 cells were kept undifferentiated and competent for BMP-induced signalling by subculture conditions that maintained a low density corresponding to approximately 150,000 cells per 182 cm^2^. Cells were split every other day when reaching 30% to 40% confluency and not used at passages higher than 20. Seeding in higher densities such as required for scratch wound healing was performed 12 hours prior to the experiment. C2C12 cells were transfected 48 hours prior to seeding in six-well plates with 0.5 to 3 μg plasmid DNA or 50nM siRNA (Dharmacon, GE Healthcare, Lafayette, CO, USA) (see Additional file 8: Table T1) using Lipofectamine2000 and Lipofectamine RNAiMAX (Invitrogen, Carlsbad, CA, USA) according to manufacturer’s instructions. HEK293T cells were transfected using polyethyleneimine and expanded in high glucose (4,500 mg/l glucose) DMEM, 48 hours prior to experiment. All experiments requiring BMP2 stimulation were conducted after 6 hours starvation in DMEM without serum. Cells were grown on uncoated cell culture plastic unless stated otherwise.

### Expression plasmids

The plasmids encoding human BMPRII-LF-HA or mouse BMPRIb-HA were described previously [20,62,63]. Single point mutations used to generate kinase dead receptors were generated by cyclic mutagenesis PCR as described in [64]. The construct encoding N-terminal flag-tagged p55γ was generated by cloning the full-length open reading frame of mouse p55γ into the TOPO-TA vector (Invitrogen, Carlsbad, CA, USA) before ligation via EcoRI/NotI into pcDNA3.1 basic. Cloning primers used in this paper are available upon request. The construct encoding HA-tagged p85α was a kind gift from Bart Vanhaesebroeck (QMUL, London, UK). The construct encoding GFP-tagged PH-domain of Akt was a kind gift from Kerstin Danker (Charité Berlin, Germany). All constructs were verified by DNA sequencing.

### Immunoprecipitation assays

Immunoprecipitation of expressed proteins from HEK293T cells was performed using a modified radio-immunoprecipitation assay buffer containing 0.5% (w/v) sodium dodecyl sulphate and 0.1% Nonidet P-40. Immunoprecipitation from C2C12 cell extracts was performed using a modified radio-immunoprecipitation assay with 0.1% sodium dodecyl sulphate and 0.5% Nonidet P-40. A detailed description of the immunoprecipitation and immunoblotting procedures can be found in Additional file 7. PIP bead assay was purchased from Echelon Bioscience and precipitation was performed according to manufacturer’s instructions.

### Mass spectrometry

Identification of p55γ binding to GST-BMPRII was performed as described in [20]. PIP bead-binding proteins were identified by matrix-assisted laser desorption ionisation-time of flight mass spectrometry-based peptide mass fingerprinting as described previously [65].

### Scratch wound healing

The scratch wound healing assay was performed using cell culture inserts (ibidi GmbH) according to the manufacturer’s instructions on uncoated tissue culture plastic. A detailed description of the procedure can be found in Additional file 7. The rate of cell migration was measured by quantifying the intensity translocation values for three independent biological replicates per condition using a selective mask filter (Slidebook).

### Boyden chamber assay

The assay was performed in a similar manner to [10] with a detailed description of the procedure in Additional file 7.

### Chemotaxis assays

Two-dimensional chemotaxis was assayed using the μ-slide chemotaxis chamber system (ibidi GmbH, Martinsried, Germany) according to accompanying instructions with the following modifications: 1 day prior to seeding, chambers were coated with 0.5% gelatin solution in humidified atmosphere washed for 1 hour and dried at 37°C. Pictures were taken using a 4× objective in bright field modus. Measurements were performed using an automated sample table mounted on an Axiovert 200 M (Carl Zeiss, Jena, Germany) in combination with Axiovision Mark&Find tool. Manual cell tracking was performed using the open source ImageJ plugin Manual tracking v2.0.

### Immunofluorescence and live cell imaging

For detection of fluorescent signals, we used the Alexa-conjugated secondary antibody system (Invitrogen, Carlsbad, CA, USA) and an inverted fluorescence Axiovert 200 microscope (Carl Zeiss, Jena, Germany) equipped with a live cell imaging heating and CO_2_ chamber mounted to a CoolSnapHQ CCD camera (Roper Scientific, Martinsried, Germany). Confocal images were taken using a Zeiss LSM519 laser scanning confocal using 63× magnification Plan Apochromat objective. A detailed description is provided in Additional file 7.

### Statistics and bioinformatics

Detailed information and description of statistical analysis on co-localisation studies, intensity translocation values, western blot quantification, used databases and artwork programmes is provided in Additional file 7.

We provide an inventory of supplemental information, supplemental experimental procedures, supplemental information and supplemental references (Additional file 7).

## Abbreviations

BISC: BMP-induced signalling complex; BMP2: Bone morphogenetic protein 2; BMPRI/II: Bone morphogenetic protein receptor type I/II; BMPRII-LF: BMP receptor type II-long form; BMPRII-SF: BMP receptor type II-short form; CLASPs: Cytoplasmic linker associated proteins; DiI: Fluorescent lipophilic cationic indocarbocyanine dye I; DiO: Fluorescent lipophilic cationic indocarbocyanine dye O; GST: Glutathione S-transferase; HA-tag: Human influenza hemagglutinin-tag; iSH2: Inter-Src homology 2 domain; p110α: p110 catalytic subunit p110 alpha; F-actin: Filamentous actin; p55γ: PI3K regulatory subunit p55 gamma; PCP: Planar cell polarity; PDK1: 3-phosphoinositide-dependent kinase-1; PH: Pleckstrin homology domain; PHLDB2 (also known as LL5β): Pleckstrin homology-like domain family B member 2; PI3K: Phosphatidylinositol-4,5-bisphosphate 3-kinase; PIP2: Phosphatidylinositol 4,5-bisphosphate; PIP3: Phosphatidylinositol (3,4,5)-trisphosphate; pSmad1: Phospho-Smad1; pTyr: Phospho-tyrosine; SH2: Src homology 2 domain; TC: Truncation; TGF-β: Transforming growth factor beta; wt: Wild type.

## Competing interests

The authors declare that they have no competing interests.

## Authors’ contributions

CH and PK designed the experiments. CH, AB and AD performed experiments. AB and IL provided computational analysis, CW performed mass spectrometry and JHB provided valuable discussion. CH and PK wrote the manuscript. All authors read and approved the final manuscript.

## Supplementary Material

Additional file 1: Figure S1 Antibody validation, quantification of co-localisation and test for BMP2 dependent tyrosine phosphorylation of endogenous BMPRII. Click here for file

Additional file 2: Figure S2 BMPRI does not co-immunoprecipitate with p55γ. Click here for file

Additional file 3: Figure S3 Knock-down efficiency of si-p55γ. Click here for file

Additional file 4: Figure S4 Wortmannin blocks BMP2 induced PI3K-Akt signalling. Click here for file

Additional file 5: Figure S5 Effect of small molecule inhibitors on signalling, PH-Akt-GFP translocation, phospho-Akt/phospho-PDK1 and BMPRII localisation. Click here for file

Additional file 6 Movie. Click here for file

Additional file 7 Inventory of supplemental information, supplemental experimental procedures, supplemental references. Click here for file

Additional file 8: Table T1 siRNA oligo sequences (Dharmacon). Click here for file
