# Supplementary material for: BMP2-induced chemotaxis requires PI3K p55γ/p110α-dependent phosphatidylinositol (3,4,5)-triphosphate production and LL5β recruitment at the cytocortex
Source: BMC Biol. 2014 May 30;12:43. doi: 10.1186/1741-7007-12-43 (PMC4071339; doi:10.1186/1741-7007-12-43)
Supplement: Additional file 1: Figure S1 — Antibody validation, quantification of co-localisation and test for BMP2 dependent tyrosine phosphorylation of endogenous BMPRII. [file 1741-7007-12-43-S1.pdf]

Additional File 1: Figure S1 (related to figure 2)

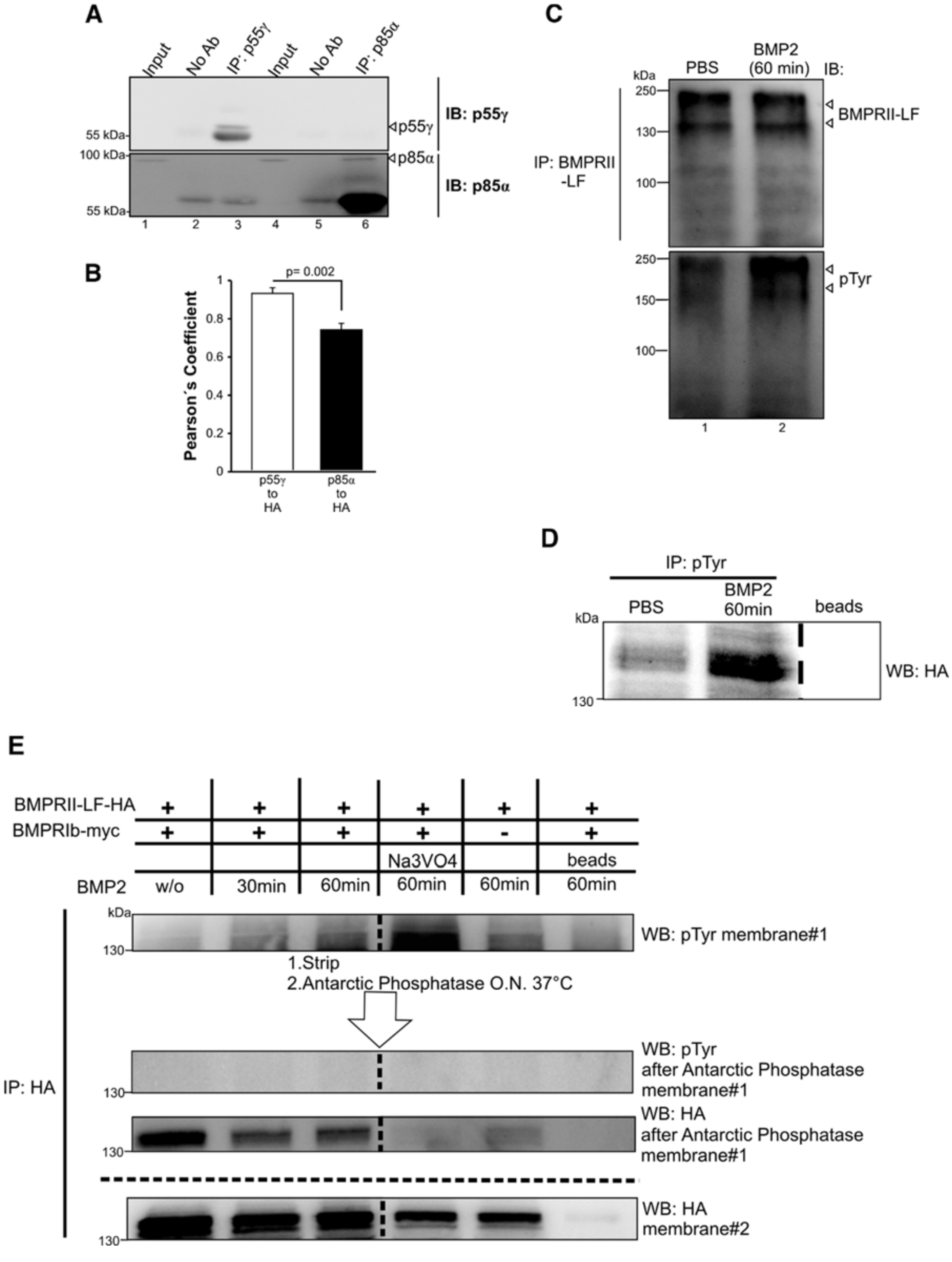

Figure S1. Antibody validation, quantification of co-localisation and test for BMP2 dependent tyrosine phosphorylation of endogenous BMPRII. (A) Validation of antibody specificity for p55γ and p85α subunits. C2C12 cell total cell lysates and extracts of immunoprecipitated p55γ and p85α were immunoblotted and incubated with p85α (upper panel) and p55γ (lower panel) antibodies showing that each antibody does precipitate and detect class Ia PI3K regulatory isoforms specifically. The molecular weights are indicated (arrowhead). (**B)** Bar diagram summarising Pearson's correlation coefficients from analysis of co-localisation p55γ and p85α with BMPRII-LF-HA. Bars represent Pearson's correlation coefficients for fluorescence intensity signals of p55γ and p85α to anti-HA. Error bars represent S.D. from 10 regions of interest in protrusions of 3 biological replicates. Student´s t-test p-values are indicated. **(C)** BMP2-induced tyrosine phosphorylation of endogenous BMPRII-LF. Immunoprecipitation of endogenous BMPRII-LF from C2C12 cell lysates showing basal and BMP2 [10nM] -induced tyrosine phosphorylation of BMPRII-LF. Arrowheads indicate the molecular weight of endogenous BMPRII-LF (upper blot), which was subjected to a stripping procedure and reblotted with pan specific pTyr antibody (lower blot). **(D)** BMP2-induced tyrosine phosphorylation of overexpressed BMPRII-LF- HA. Immunoprecipitation upon 60 minutes BMP2 [10nM] stimulation of HEK293T cells using anti-pTyr antibody, followed by anti-HA western blot compared to a PBS treated control. **(E)** Test for pTyr specificity of the used antibody. To confirm phospho- specificity of the tyrosine antibody, HEK293T cells were transfected with BMPRII-LF-HA and/or not with BMPRIb. Cells were starved for 6 hours and treated with 10nM BMP2 for indicated time. Pre-treatment with 1mM of tyrosine-phosphatase inhibitor Sodium-Orthovanadate was performed 1 hour prior to stimulation and precipitation via anti-HA antibody. The precipitates were separated on SDS-PAGE and blotted with anti-pTyr antibody. The membrane was then stripped and then incubated with 100 Units Antarctic Phosphatase (NEB #M0289) O.N. at 37°C to dephosphorylated proteins on membrane. Membrane was subsequently re-blotted with pTyr specific antibody. A pulldown control after

stripping and re-blotting proved for the precipitated BMPRII-LF. A second SDS-PAGE of precipitated BMPRII-LF from the same experiment was loaded to exclude protein waste due to stripping and excessive washing steps. Dotted lines indicate exclusion of irrelevant lanes.
