# Supplementary material for: BMP2-induced chemotaxis requires PI3K p55γ/p110α-dependent phosphatidylinositol (3,4,5)-triphosphate production and LL5β recruitment at the cytocortex
Source: BMC Biol. 2014 May 30;12:43. doi: 10.1186/1741-7007-12-43 (PMC4071339; doi:10.1186/1741-7007-12-43)
Supplement: Additional file 2: Figure S2 — BMPRI does not co-immunoprecipitate with p55γ. [file 1741-7007-12-43-S2.pdf]

Additional File 2: Figure S2 (related to figure 3)

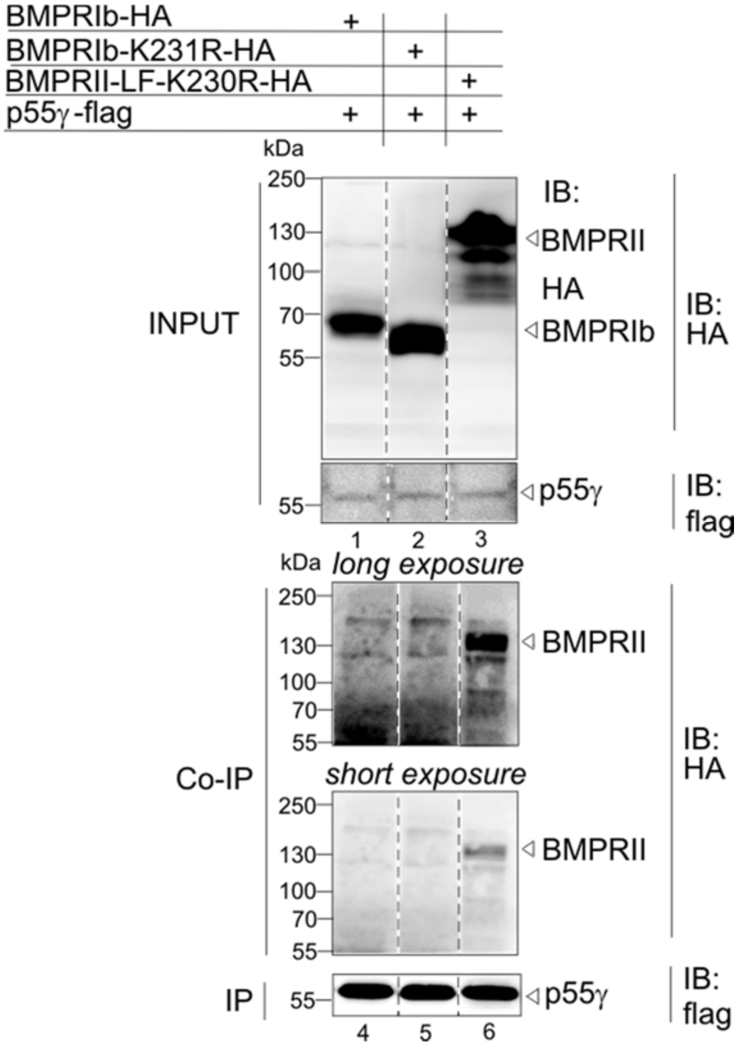

Figure S2. BMPRI does not co-immunoprecipitate with p55γ. Co-IP in HEK293T cells transfected with HA tagged BMPRIb, respective kinase dead mutant (BMPR1b-K231R-HA) or BMPRII-LF-HA, together with flag-tagged p55γ. Dotted lines indicate exclusion of nonrelevant conditions from the same blot. Upper panel shows no interaction of HA-tagged BMPRIb or BMPRIb-K231R at short- and long-exposure times.(lanes 1 and 2, expected MW of co-immunoprecipitated BMPRIb-HA: ~65kDa). The interaction of flag-tagged p55γ with BMPRII-LF-HA (lane 3, ~160kDa) was used as a positive control.
