# Supplementary material for: BMP2-induced chemotaxis requires PI3K p55γ/p110α-dependent phosphatidylinositol (3,4,5)-triphosphate production and LL5β recruitment at the cytocortex
Source: BMC Biol. 2014 May 30;12:43. doi: 10.1186/1741-7007-12-43 (PMC4071339; doi:10.1186/1741-7007-12-43)
Supplement: Additional file 3: Figure S3 — Knock-down efficiency of si-p55γ. [file 1741-7007-12-43-S3.pdf]

Additional File 3: Figure S3 (related to figure 4 and 5)

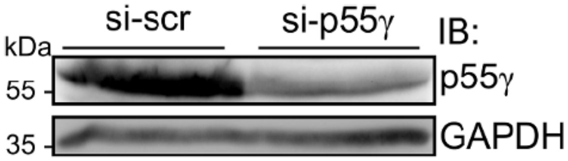

Figure S3. Knock-down efficiency of si-p55γ. Western Blot analysis of C2C12 cell lysates showing endogenous p55γ protein after 48 hours upon siRNA mediated knock-down. Cells were transfected with either scrambled control siRNA (si-scr) or with siRNA specifically targeting p55γ (si-p55γ).
