# Supplementary material for: BMP2-induced chemotaxis requires PI3K p55γ/p110α-dependent phosphatidylinositol (3,4,5)-triphosphate production and LL5β recruitment at the cytocortex
Source: BMC Biol. 2014 May 30;12:43. doi: 10.1186/1741-7007-12-43 (PMC4071339; doi:10.1186/1741-7007-12-43)
Supplement: Additional file 4: Figure S4 — Wortmannin blocks BMP2 induced PI3K-Akt signalling. [file 1741-7007-12-43-S4.pdf]

Additional File 4: Figure S4 (related to figure 4)

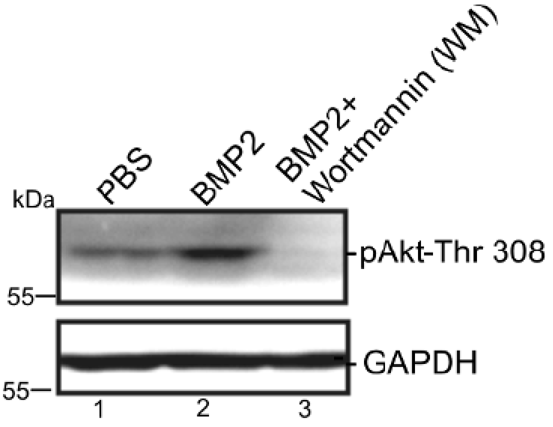

Figure S4. Wortmannin blocks BMP2 induced PI3K-Akt signalling. Effect of Wortmannin pre-treatment on BMP2-induced PI3K signaling. C2C12 cells were pre-treated for 1 hour with Wortmannin [25nM] and BMP2 induced Akt Thr 308 phosphorlyation was assayed upon 60 minutes stimulation.
