# Supplementary material for: BMP2-induced chemotaxis requires PI3K p55γ/p110α-dependent phosphatidylinositol (3,4,5)-triphosphate production and LL5β recruitment at the cytocortex
Source: BMC Biol. 2014 May 30;12:43. doi: 10.1186/1741-7007-12-43 (PMC4071339; doi:10.1186/1741-7007-12-43)
Supplement: Additional file 5: Figure S5 — Effect of small molecule inhibitors on signalling, PH-Akt-GFP translocation, phospho-Akt/phospho-PDK1 and BMPRII localisation. [file 1741-7007-12-43-S5.pdf]

Additional file 5: Figure S5 related to Figure 5

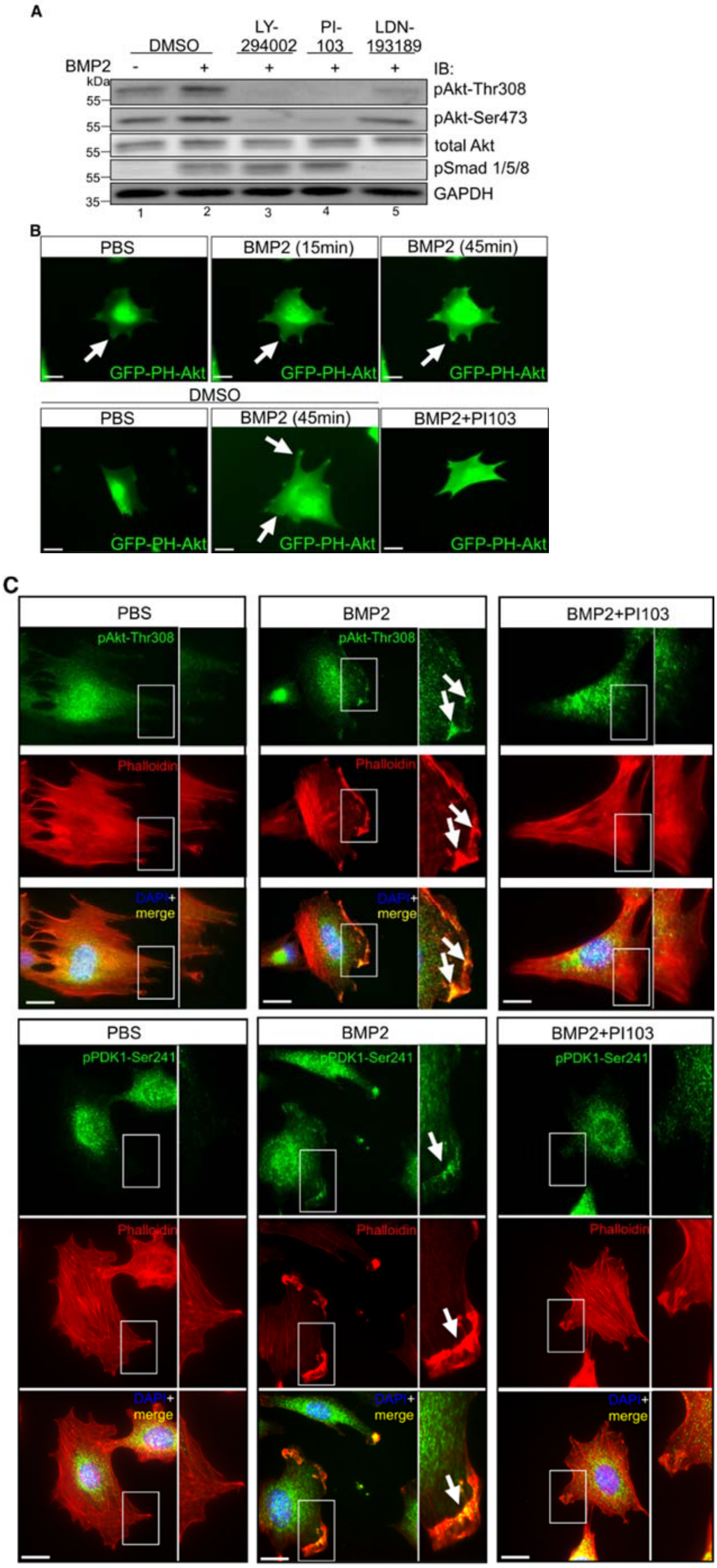

D

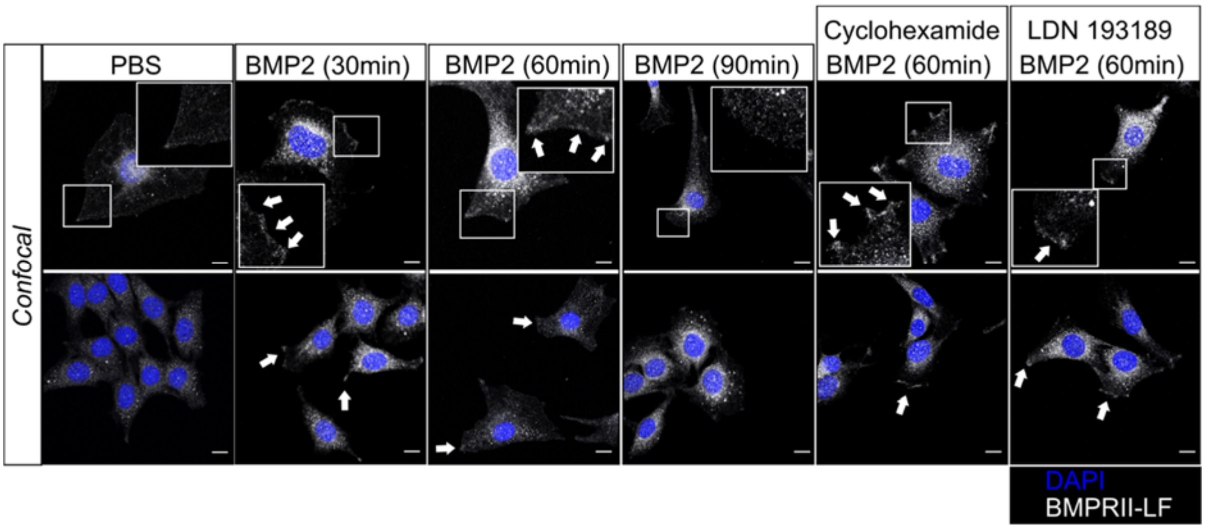

Figure S5. Effect of small molecule inhibitors on signalling, PH-Akt-GFP translocation, phospho-Akt/phospho-PDK1 and BMPRII localisation. (A) Effect of PI3K specific and BMP type I receptor specific inhibitors on BMP2 signaling. Western Blot analysis showing BMP2-dependent phosphorylation of Akt-Thr303, Akt-Ser472 and Smad1/5/8. Cells were pre-treated for 1 hour with inhibitors LY-294002 [10µM], PI103 [8nM] and LDN-193189 [0.5µM] and stimulated with BMP2 [10nM] for 60 minutes. The effect of small molecule inhibition on BMP2 induced signalling was compared to a DMSO treated control. **(B)** BMP2-dependent GFP-PH-Akt translocation. Immunofluorescence of C2C12 cells transfected with GFP-PH-Akt. Upper panel shows stills of a 45 minutes time laps series of BMP2 [10nM] stimulated C2C12 cells transfected with GFP-tagged PH domain of Akt. White arrowheads point towards increased GFP signal accumulation. The lower panel shows GFP-PH-Akt translocation upon 45 minutes BMP2 stimulation [10nM] compared to GFP-PH-Akt translocation when cells were pre-treated with PI103 [8nM]. White arrowheads point towards areas of intense GFP signal. Scale bars represent 20 µm. **(C)** BMP2 induces phospho Akt and phospho PDK1 at the leading edge. Immunofluorescence showing localization of phosho-Akt Thr308 and phospho PDK1-Ser241 together with F-actin in C2C12 cells upon BMP2 [10nM] stimulation for 60 minutes. Protrusive regions with cortical actin are magnified. BMP2-induced localization of cortical actin and phospho-Akt Thr308 as well as phospho-PDK1 were compared to cells pre-treated with the class Ia PI3K selective inhibitor PI103 [8nM]. Arrowheads point towards co-localization of phospho-Akt Thr308 and cortical actin. Scale bars represent 20µm. **(D)** Confocal analysis of the localisation of endogenous BMPRII-LF in C2C12 cells upon 10nM BMP2 treatment for indicated time using an antibody which detects the c-terminal tail of BMPRII-LF. (Cell Signaling Tec. #6979). Cyclohexamide [10mg/ml] treatment was performed 6 hours and

0.5µM LDN193189 1 hour prior to stimulation. Arrows indicate for ruffle associated endogenous BMPRII. Scale bars indicate 10µM.
