# Supplementary material for: BMP2-induced chemotaxis requires PI3K p55γ/p110α-dependent phosphatidylinositol (3,4,5)-triphosphate production and LL5β recruitment at the cytocortex
Source: BMC Biol. 2014 May 30;12:43. doi: 10.1186/1741-7007-12-43 (PMC4071339; doi:10.1186/1741-7007-12-43)
Supplement: Additional file 7 — Inventory of supplemental information, supplemental experimental procedures, supplemental references. [file 1741-7007-12-43-S7.pdf]

Inventory of supplemental information, supplemental experimental procedures and supplemental references

1. Supplemental data files provided with this manuscript

Additional file 1: Figure S1 related to Figure 2

Antibody validation, quantification of co-localisation and test for BMP2 dependent tyrosine phosphorylation of endogenous BMPRII.

Additional file 2: Figure S2 related to Figure 3

BMPRI does not co-immunoprecipitate with p55γ.

Additional file 3: Figure S3 related to Figure 4

Knock-down efficiency of si-p55γ.

Additional file 4: Figure S4 related to Figure 4

Wortmannin blocks BMP2 induced PI3K-Akt signalling.

Additional file 5: Figure S5 related to Figure 5

Effect of small molecule inhibitors on signalling, PH-Akt-GFP translocation, phospho-Akt/phospho-PDK1 and BMPRII localisation.

Additional file 6: Movie related to Figure 5C

Additional file 7: Inventory of supplemental information, supplemental experimental procedures, supplemental references.

Additional file 8: Tabel T1, siRNA oligo sequences

2. Supplemental experimental procedures (below)

- **Antibodies and Western Blotting**
- **Phospho-protein assay**
- **Co- Immunoprecipitation**
- **Immunofluorescence microscopy**
- **Life cell imaging**
- **PI3K activity ELISA**
- **Scratch wound healing**
- **Boyden Chamber Assay**
- **Statistics, bioinformatic analysis and artwork**

3. Supplemental references (below)

References refer to the supplemental experimental procedures section.

2. Supplemental experimental procedures

Antibodies and Western Blotting

Antibodies to BMPRII (G17) (sc-5682), p55γ (E9) (sc-376615), PHLDB2 (N15) (sc-99590) and GFP (sc-9996) were purchased from Santa Cruz Biotechnology, Inc. The antibodies to p110α (#4294), pAkt-Thr308 (#2965), pAkt-Ser407 (#4060), pan Akt (#4691), pP70S6K-Thr421/Ser424 (#9204), pGSK3β-Ser9 (#9323), pPTEN-Ser380 (#9551), pSmad1/5/8 (#9511), Smad1 (#6944), p85α (#4292), pPI3K p85-(Tyr458)/p55 (Tyr199) (#4228), GAPDH (#2118), Syntaxin 6 (#2869) and pan p-Tyr (# 9416) and c-terminal tail of BMPRII-LF (for IF) (#6979) were purchased from Cell Signaling Technology. The antibody to p-p38 was purchased from Promega. Antibodies Flag-M2 (F1604) and HA7 (H3663) were purchased from Sigma Aldrich. The anti-PIP_3 antibody (#Z-G345) was purchased from Echelon Bioscience. Protein lysates were subjected to SDS-PAGE and transferred to PVDF membranes by Western blotting. Membranes were blocked for 1 h in 0.1% TBS-T containing 5% skim milk, washed three times in 0.1% TBS-T and incubated with indicated primary antibodies overnight at 4°C following manufacturer's recommendations. For HRP-based detection, goat anti-mouse, goat anti-rat or donkey anti-goat HRP conjugates were used. Detection of adsorbed HRP-coupled secondary antibodies was performed by ECL reaction. HRP signals were detected using a CCD-based detection system (Vilber Lourmat). Membranes subjected to a second round of immunoblotting were stripped with stripping buffer (62.5mM Tris-HCL pH 6.8, 2%SDS, 100mM β-mercaptoethanol) and incubated at 55°C for 30 minutes with mild shaking before excessive washing with tab water and re-blocking.

Phospho-protein assay

Cells were seeded in 6-well plates at a density of $0.5x10^5$ cells per well. After 48 hours, cells were starved for 6 hours and incubated in the absence or presence of 10nM ligand for the indicated times. Inhibitor pre-treatments were performed for 1 hour prior to stimulation with complete exchange of the starvation medium. Cells were lysed in 1x SDS-sample buffer, scraped, boiled and applied on 10% PAA gels for Western blot analysis. To confirm phospho-specificity an Antarctic Phosphatase treatment of membrane was carried out using 100 units of Antarctic phosphatase (NEB, M0289) in corresponding 1X buffer O.N. at 37°C.

Co- Immunoprecipitation

Modified RIPA for immunoprecipitation from expressed proteins (HEK293T) contained 50mM Tris, 150mM sodium chloride, 0.5% sodium dodecyl sulphate (SDS), 0.1% Nonidet P-40 and 1mM phenylmethylsulfonylfluorid, 2mM sodium orthovanadate, 50mM sodium fluoride, 200mM sodium pyrophosphate and 1x EDTA free protease inhibitor cocktail (Roche). Immunoprecipitation from C2C12 cell extracts was performed using modified RIPA with 0.1% SDS and 0.5% Nonidet P-40. In brief, cells were washed with ice cold PBS and scraped. Cell lysis was performed for 30 minutes in a rotation wheel at 4°C. Cell lysates were passed through a 26-gauge needle and non-soluble fraction was removed by pre-clearing for 30 minutes centrifugation at 16,000g at 4°C. Supernatant was subjected to incubation with 1-3 µg of primary antibody for 2.5 hours. Immunocomplexes were precipitated with Protein A (GE Healthcare)/Protein G (Sigma Aldrich) sepharose slurry (50:50) for 2.5 hours. Beads were washed 3 times in fresh lysis buffer including inhibitors. Elution was achieved by 2x SDS sample buffer. Prior to loading on SDS-PAGE samples were boiled for 15 minutes and 1mM dithiothreitol was added.

Immunofluorescence microscopy

Cells ($1x10^4$) for immunofluorescence staining were plated on sterile glass coverslips placed in 6-well plates unless stated otherwise. After 48 hours cells were starved and subsequently stimulated in DMEM without FBS for the indicated times. Immunofluorescence staining was performed as described in (Schwappacher R. et al. EMBO 2009). In brief, cells were fixed in 4% paraformaldehyde and permeabilized in 0.5% Triton-X-100 (or 0.5% Saponin in case of PIP_3 stainings) for 15 minutes at room temperature. After blocking with 3% BSA, cells were stained using the indicated primary antibodies, and Alexa 488 or 595 conjugated secondary antibodies (Invitrogen). Phalloidin was purchased from (Invitrogen). Pictures were taken using an inverted fluorescence microscope Axiovert 200 mounted to a 40x Zeiss plan apochromat objective (Carl Zeiss Microimaging). Images were analysed using linear BestFit option of Zeiss Axiovision (Carl Zeiss Microimaging) and Photoshop software (Adobe). For confocal analysis a Zeiss Laser Scanning microscope 510 in combination with ZEN software and an 63X Plan apochromat objective was used.

Life cell imaging

Imaging of single cells was performed after seeding on uncoated glass bottom dishes (MatTek Corporation) within a heat and CO_2 controlled Life Cell Imaging chamber (ibidi GmbH)

mounted on a Axiovert 200M inverse microscope (Zeiss). Long-term image stacks were gained by application of axiovision (Zeiss) digital autofocus correction. Growth factor application to imaged cells on the heating stage was performed using the TransferMan micromanipulator system (Eppendorf AG).

PI3K activity ELISA

Lipid kinase activity was tested using the PI3-Kinase Activity ELISA kit (K-1000s) purchased from Echelon Bioscience (Salt Lake City) essentially as described by (Greger et al., 2007). Briefly, p55γ and p85 were immunoprecipitated using specific antibodies and subjected to an in vitro kinase reaction using PtdIns(4,5)P_2 as a substrate. The presence of p110α catalytic subunit for each precipitated sample was confirmed by Western blotting. Reaction products were first mixed and incubated with a PI(3,4,5)P3 detector protein, then added to the PI(3,4,5)P3-coated microplate for competitive binding. A peroxidase- linked secondary detector and colorimetric detection at 450nm using a TECAN plate reader was used to detect PI(3,4,5)P3 detector binding to the plate. The colorimetric signal is inversely proportional to the amount of produced PI (3,4,5)P3 from the immunoprecipitates.

Scratch wound healing

The scratch wound healing assay was performed using cell culture inserts (ibidi GmbH) according to manufactures instructions on uncoated tissue culture plastic. C2C12 cells were seeded to become confluent and starved for 6 hours in 0.5% FCS DMEM. 2 hours prior to insert removal and stimulation, cells were incubated with 10 µg/ml mitomycin C to block cell proliferation. Membrane staining of siRNA-transfected cells was performed using DiI and DiO (Life Technologies GmbH) according to the accompanying protocol. For the respective siRNA oligonucleotide sequence, please see Table S2.

After insert removal, the wound closure was allowed to proceed in the presence or absence of 10nM BMP2 and imaged by fluorescence and phase-contrast microscopy. The filter measured the intensity of the fluorescence signals within the invaded area with respect to the initial wound area. Trajectories of single cell migration were gained by manual adjustment of mean centre of intensity for individual cells. Cell movements were tracked by analysing image stacks consisting of 9 time-lapse images. Resulting trajectories were plotted on the phase contrast image corresponding to the end time-point.

Boyden Chamber Assay

The assay was performed as described previously (Gamell *et al.*, 2008) with the following modifications. Briefly, $5x10^4$ of siRNA-transfected C2C12 cells were starved for 6 hours, trypsinised and seeded onto cell culture inserts with polycarbonate membrane with 8 µm pore size (Thermo Fisher Scientific) in 0.5ml of media containing 0.2% FCS and inserts were placed in the 24-well plate filled with 0.5ml of the same media per well. Plates were placed in the incubator to let the cells adhere to the membrane. After 1 hour the medium in the lower chamber was replaced with media containing 10nM BMP2 and cells were left for 5 hours in the incubator for migration. Next, cells were fixed with 4% PFA, the membranes were cut out from the inserts, nuclei were stained with DAPI and membranes were mounted on the microscopy slides. Cell migration was analysed using a fluorescence microscope by taking 7 pictures with 5x magnification at equal positions of each membrane in triplicates per condition. Bars represent the number of counted cells with values expressed as means +/- S.D. of three independent experiments.

Statistics, bioinformatic analysis and artwork

Quantitative analysis of scratch wound healing was performed using Slidebook 5 (Intelligent Imaging Innovations Inc.) For single-colour wound healing, translocation into the wound was analysed by application of a mask on images taken at start time point as a single rectangular mask. The mask was then transferred to images taken at end time point and the sum intensity of the fluorescent signal localized within the wound area was used as quantification parameter. The sum fluorescence intensity localized within the wound compared to the sum intensity of the sample produced the relative intensity translocation ratio. A selective mask for fluorescence signal was created by setting low- and high-gate values. The fluorescence signal was also determined in images at start time-point and the sum intensity value localized within the wound was used as baseline for the end time-point. Area within the wound region was used to determine background fluorescence.

For double colour analysis, the relative intensity translocation ratio described above was compared between differently stained cells.

For statistics, intensity values of 19 samples were divided in two groups according to sum intensity values to compensate for sample inhomogeneity due to the different transfection efficacy between biological replicates. Intensity values of samples belonging to group with higher sum intensity were normalized to a value from the group with lower sum intensity. The means +/- S.D. were calculated.

Comparison of multiple groups was done by one-way analysis of variance (ANOVA) with post-hoc Bonferroni test. All pair wise comparisons were done by two-tailed Student's t-test. Statistical calculations were performed using SigmaPlot software (Systat) and *p*-values are indicated in given figures with *p*-values of <0.05 considered as statistically significant. Quantification of Western blots was performed using Bio1D software (Vilber Lourmat) or NIH Image J. Image analysis was performed using WCIF Image J plugin and Pearsons´s coefficient was determined to quantify co-localisation. All measurements were performed in at least three independent experiments. The means +/- S.D. were calculated. The phospho-motif scan for SH2 domain binding of PI3K regulatory subunit to phosphorylated tyrosine residues in BMPRII-LF was performed using ScanSite motif scan applying medium stringency filter. Artwork was done using COREL Draw 14.0 (Corel Corporation).

3. Supplemental references

Bretones G, Acosta JC, Caraballo JM, Ferrándiz N, Gómez-Casares MT, Albajar M, Blanco R, Ruiz P, Hung WC, Albero MP, Perez-Roger I, León J. (2011) SKP2 oncogene is a direct MYC target gene and MYC down-regulates p27(KIP1) through SKP2 in human leukemia cells. J Biol Chem. 286, 9815-25

Gamell, C., Osses, N., Bartrons, R., Ruckle, T., Camps, M., Rosa, J.L. and Ventura, F. (2008) BMP2 induction of actin cytoskeleton reorganization and cell migration requires PI3-kinase and Cdc42 activity. J Cell Sci, 121, 3960-3970.

Geering, B., Cutillas, P.R., Nock, G., Gharbi, S.I. and Vanhaesebroeck, B. (2007) Class IA phosphoinositide 3-kinases are obligate p85-p110 heterodimers. Proc Natl Acad Sci U S A, 104, 7809-7814.

Greger JG, Fursov N, Cooch N, McLarney S, Freedman LP, Edwards DP, and Cheskis BJ (2007) Phosphorylation of MNAR Promotes Estrogen Activation of Phosphatidylinositol 3-Kinase. Mol Cell Biol, 5, 1904–1913.

Schwappacher R, Weiske J, Heining E, Ezerski V, Marom B, Henis YI, Huber O, Knaus P. (2009) Novel crosstalk to BMP signalling: cGMP-dependent kinase I modulates BMP receptor and Smad activity. EMBO J, 28, 1537-50.

Ueki, K., Fruman, D.A., Yballe, C.M., Fasshauer, M., Klein, J., Asano, T., Cantley, L.C. and Kahn, C.R. (2003) Positive and negative roles of p85 alpha and p85 beta regulatory subunits of phosphoinositide 3-kinase in insulin signaling. J Biol Chem, 278, 48453-48466.
