# Supplementary material for: BMP2-induced chemotaxis requires PI3K p55γ/p110α-dependent phosphatidylinositol (3,4,5)-triphosphate production and LL5β recruitment at the cytocortex
Source: BMC Biol. 2014 May 30;12:43. doi: 10.1186/1741-7007-12-43 (PMC4071339; doi:10.1186/1741-7007-12-43)
Supplement: Additional file 8: Table T1 — siRNA oligo sequences (Dharmacon). [file 1741-7007-12-43-S8.pdf]

Additional File 8: Table T1

si-p55γ		AAGGAGAUCGAGCGAAUUA
si-LL5β		AGAGGAAGGAUUUGUGGUA

siRNA oligo sequences (Dharmacon)
